# Gender differences in self-view and desired salaries: A study on online recruitment website users in China

**DOI:** 10.1371/journal.pone.0210072

**Published:** 2019-01-10

**Authors:** Xiaoqi Zhang, Yanqiao Zheng

**Affiliations:** School of Finance, Zhejiang University of Finance and Economics, Hangzhou, China; Middlesex University, UNITED KINGDOM

## Abstract

One explanation for the gender pay differences in labor markets is that women propose lower desired salaries. By using an actual job seeking resume database and applying text mining techniques, we are able to observe both the extent of gender differences in desired salaries and job-related self-view. We find gender differences in global self-view favoring females, and in some domain-specific self-view favoring males. Previous findings of disadvantaged groups having levels of self-view at least as high as those of advantaged groups lend credibility to our findings. Moreover, we argue that the differences in global self-view favoring females may be related to the theories of “belief flipping”, since women in our sample of online-recruitment markets are distinct from the general population, with on average 15.2 years of education and 8.99 years of work experience, due to self-selection. In addition, we find that women do propose lower desired salary than men, after controlling for various factors such as human capital, marital status, industries. We further investigate the role of self-view and find it contributes to explain desired salaries, with modest mediator effect but little moderator effect on gender differences in desired salaries.

## Introduction

Around the world, women earn less than men [1]. For example, in the U.S., the median weekly earnings of women above 25 years of age were about 81.3% of those of their male counterparts in 2017 [2]. The reasons are manifold: gender differences in human capital; gender segregation in school majors and in labor markets; maternal responsibilities; gender discrimination; gender differences in preferences for competition and security, etc. [3–6].

There is another important branch of research suggesting a different determinant of gender differences in labor markets: men propose higher desired salary and are more likely to engage in salary negotiations than women [7–11]. For example, [9] report survey data on 435 undergraduate students suggesting that female students have an 8.3% lower desired career-entry salary and 33% lower desired career-peak salary than male students. Relatedly, [10] observe that in a laboratory experiment on MBA students, single female students reported lower desired salaries and willingness to travel and work long hours on a real-stakes placement questionnaire, in order to be seen as more acceptable in marriage markets. Finally, [11] find from a natural field experiment that when there is no explicit statement that wages are negotiable, men are more likely to negotiate for a higher wage, whereas women are more likely to signal their willingness to work for a lower wage.

In this study, we depart from traditional investigation by using a large database of job seeking resumes in actual labor markets. We use data from a major online recruitment website in China. There are several advantages in using actual labor market data. First, the sample size is adequate to overcome some statistical issues. Second, the sample population is from a wide range of labor forces who have already accumulated some real-world work experience, instead of student samples utilized by most previous research. Last but most importantly, subjective items such as desired salary and self-view can vary substantially under different circumstances, as documented by [10–12], etc. Our study on actual job-seeking resumes could offer new evidence on gender differences in labor markets.

Another contribution of this study is the investigation of the role of self-view in determining salary differences and its possible mediator and moderator effects. Thanks to text data mining techniques, we are able to extract key words and quantify the dimensions of self-view from its text-format information. The psychology and sociology literatures are rich with self-related theories and sex-based stereotypes but still lack connections from theoretical investigations to everyday problems [13–14]. In this way, our study moves the exploration of effects of self-view from the lab to actual labor markets.

Our results suggest that women indeed propose lower desired salary than men, controlling for human capital, marital status, work status, enterprise type, and industry fixed. While women even have slight advantage in education in terms of education years, elite university degrees and certificates held, they fall behind in work experience with shorter years in labor markets, less willingness to change jobs for promotion, and higher probability to compromise for marriage. These results square well with laboratory evidence [8,10,12].

We find gender differences in global self-view favoring females, and in some domain-specific self-view favoring males. To reconcile some differences with the existing literature, we perform the gender differences of self-view across different groups, based on whether they are fresh graduates, whether they are in female-dominated industries, and across groups of different marital status, and length of work experience. Finally, we find self-view measures are good contributors to explaining desired salaries, with modest mediator effect but little moderator effect on gender differences in desired salaries.

We view these findings as a new piece of evidence on gender differences in self-view and desired salaries. Beyond providing an estimate of gender differences in desired salaries caused by human capital, marital status, employment status, etc., we connect self-view to desired salaries using text data mining techniques on job seeking resumes in actual labor markets.

## Data and preliminary observations

We use resume data from zhaopin.com, one of the largest online recruitment websites in China. The reason we choose zhaopin.com is that it provides information on desired salary, self-view, and other variables we need. Zhaopin.com was established in 1994, and its business covers a vast majority of cities in China. Its resume data contains variables such as age, education experience, work experience, job intention (including desired salary), work status (working or out of work), self-view, residence city, work place, hukou affiliation, etc. For this study, we choose a random sample that comprises job seekers of working age. Specifically, the sample consists of men are between age 18 and 65, and women between age 18 and 60, looking for a full-time job, and that relevant variables are not missing. We randomly selected 25000 resumes in June 2017, with 20593 resumes remained after cleaning, of which 10012 are men and 10581 are women. The quantitative self-view data is achieved by analyzing free-style texts of self-view using “Jieba”, a Python module dealing with Chinese word segmentation.

Unlike LinkedIn, resume data from zhaopin.com can only be seen by potential employers, and is *not* available to the public (The data is officially acquired from a database initiated by Minsheng Weekly. The database by Minsheng has a random subsample of the resume database of zhaopin.com. Minsheng Weekly is a subsidiary body held by People’s Daily. The data is accessible for research purpose, but one has to apply for permission via their official website http://www.cnbo.tv or http://www.msweekydata.com, or email address cnbotv@163.com. We complied with the terms of service for the websites from which we collected data). Therefore, the platform of zhaopin.com serves merely as a job market instead of social media or social network. This guarantees that job seekers produce their resumes without concerns about signaling undesirable traits, such as ambition, to friends or acquaintances or potential dating mates. In this way, our work may add to the literature by observing job seekers in a relatively solitary and private environment, compared to in-classroom questionnaires.

Supplemental data of average housing prices by city is extracted from the Monthly Report of Housing Market in China (June 2017) published by the Chinese Academy of Social Science, adjusted and completed with reference to fang.com and lianjia.com, two largest housing transaction websites in China.

### A. Desired salary

Job seekers are asked to fill in an online resume when they register on zhaopin.com, including a drop-down menu named “Desired Salary” as a subsection of “Job Intention”. The items in the drop-down menu include (in the unit of RMB/month): below 1000, 1000–2000, 2001–4000, 4001–6000, 6001–8000, 8001–10000, 10001–15000, 15000–25000, 25000–35000, 35000–50000, 50000–70000, 70000–100000, above 100000, and negotiation face-to-face. The distribution of the desired salaries by gender is shown in Table 1 after 98% winsorization (below 1% and above 99% reset to the boundary values). Overall, the majority of the desired salaries are between 4000 and 15000. As regard to gender difference, women occupy more than half of the population when the desired salary is below 8000, and less than half when above 8000.

**Table 1:** Distribution of desired salaries (unit: RMB/ month).

	2001–4000	4001–6000	6001–8000	8001–10000	10001–15000	15001–25000	25001–35000	35000–50000	Overall
Female	1,521	2,844	2,124	1,477	1,425	847	238	105	10,581
Male	568	1,599	1,850	1,583	1,853	1,511	649	399	10,012
Percentage female	72.81%	64.01%	53.45%	48.27%	43.47%	35.92%	26.83%	20.83%	51.38%

### B. Individual characteristics

Table 2 describes gender differences in individual characteristics. We take the mid-point value of the desired salary in each range of the drop-down menu (e.g., we take 5000 for values in the range 4001–6000), and then take the logarithm of the mid-point value to create a new variable, lnsalary. As can be seen from Table 2, women propose significantly lower desired salaries. It is worth mentioning that women spend 0.13 more years in education, have an equal proportion graduating from a “985” university and a higher proportion graduating from a “211” university, and hold on average more certificates than men (The Chinese government ranks domestic universities and classifies them as “985 Project Universities” and “211 Project Universities”. As of 2018, there are 39 universities listed in “985 Project Universities”, as the first-tier, and 112 universities listed in “211 Project Universities”, as the second-tier). Overall, it means women even have a slight advantage in education.

**Table 2:** Gender differences in individual characteristics.

Variables	Female	Male	Difference
lnsalary	8.89	9.22	-0.33***
edu-years	15.2	15.08	0.13***
grad_985	0.13	0.12	0
grad_211	0.12	0.11	0.01***
cert-num	1.17	0.97	0.20***
age	30.2	32.26	-2.06***
work-years	8.99	11.18	-2.19***
work-num	3.09	3.43	-0.35***
SOE	0.12	0.21	-0.09***
listed	0.11	0.16	-0.05***
foreign	0.13	0.15	-0.01***
private	0.44	0.53	-0.08***
married	0.24	0.34	-0.10***
unmarried	0.36	0.35	0
working	0.33	0.37	-0.03***
graduate	0.03	0.02	0.01***
satisfied	0.05	0.08	-0.03***
leave	0.58	0.53	0.05***
N	10581	10012	

Note:

Note: ***,**,* denote significance levels of 1%, 5% and 10%.

Women are 2.06 years younger than men. This is mainly due to the fact that women may quit the labor market earlier because they more often take care of the family and also enjoy a lower legal retirement age (In China, females do most, if not all, of housework., and currently the legal retirement age for female is 5 years younger than for male). This can be confirmed by the observation that female job seekers who have already left their job (variable: *leave* in Table 2), the group that might permanently quit labor markets, occupy a larger proportion than men. It is consistent with the observation that women’s working experience is 2.19 years less than men. As a result, their proportions of working at different types of enterprises, namely state-owned, listed, foreign, and private firms, are significantly lower than men’s.

As to the marriage status, it is shown that the proportion of married women is 24%, significantly lower than that of married men, which is 34%. This may have two reasons. First, married women prefer stability over job changes [15]. This can be confirmed from the observation of work status: though female job seekers as a fresh graduate (variable: *graduate*) occupy a larger proportion than men, females who are still working (variable: *working*) or even satisfied with current work (variable: *satisfied*) are less likely to seek jobs elsewhere than men. The second reason for female job seekers’ lower proportion of being married is that they choose not to disclose the marital status to avoid gender discrimination based on birth and parenting pressure, since it is optional to fill in marital status in our resume data.

## Self-view

Earlier studies on gender differences in self-view mostly use college student sample. However, the conclusion on gender differences in self-view may be a peculiarity of student samples and may not extend to other groups. Similar argument is documented by [16] on gender differences in altruistic behavior. Besides, even people of the same age group may have quite different levels of self-esteem depending on their personal attributes and the working class they are from. Our sample of working-age white-collar women may exhibit a higher level of self-view than general women at large. As is documented by [17], women of different attributes (masculine or feminine) are observed to show significantly different levels of altruism. In addition, gender differences in self-view may not be extended from lab observations to the actual labor market. Literature abounds in emphasizing the situation setting. For example, gender differences in moral judgement and honesty vary in different situations [18–19].

### A. Text quantification

Our resume data could help address the above issues. However, self-views in the resume data are textual descriptions. How to quantify those descriptions is a key in this study. The way we tackle this problem is by adopting “Jieba”, a Python Chinese word segmentation module. Then we extract the high frequency words from the pool of all self-view texts, and finally record if a high-frequency word occurs in each resume. In detail, first we use “Jieba” to segment all the words in the pooled self-view text data and sort the words according to their frequencies. We exclude general words like “I”, “work”, “firm”, preposition and conjunction words like “and”, “in”, “of”, and punctuation. The frequency ranking of the remaining words is shown in Table 3. Specifically, we classify the words into two groups: self-esteem and self-efficacy as adopted in psychology literature [20].

**Table 3:** Frequency counts of self-view words.

Self-esteem	Frequency	Self-efficacy	Frequency
responsible	4995	Team	12619
conscientious	4902	Learn	11603
active	3865	Communicate	11563
outgoing	3778	Organize	5683
optimistic	3609	Coordinate	4758
hardy	3579	Adapt	2958
reliable	3450	Deal	2738
enthusiastic	3396	Problem-solving	2611
independent	2838		
hard-working	2318	Challenge	2559
steady	2299	execute	2271
honest	1958	Stress-	2194
excellent	1955	tolerance	

Self-esteem is a judgment of personal value [20]. Global self-esteem is one of the most widely researched psychological constructs. Previous research suggests that self-esteem can interact with gender to influence the image people have of themselves receiving pay in the future [9]. Self-efficacy is an appraisal of one’s competence [21], and is also linked to gender and pay-related variables. Self-esteem and self-efficacy are distinct yet related constructs. In fact, the concept of self-esteem and self-efficacy as two mainly adopted concepts of self-view in psychology literature [9,22], which guarantees their validity as measures of self-view.

Our measures of self-esteem and self-efficacy follow the method of lexical tradition in psychology literature. There are two prominent systems for measuring personal trait, one derived from the lexical tradition and one from the questionnaire tradition, namely natural language adjectives and theoretically based personality questionnaires [23]. The questionnaires method may show redundancy of questions and little resemblance to each other due to remarkably diversification of underlying theories and hopelessness to identify basic dimensions. In this sense, the advantage of natural language approach is that it could identify a few hundred adjectives with some confidence of their representation of ordinary social language.

**Fig 1:**
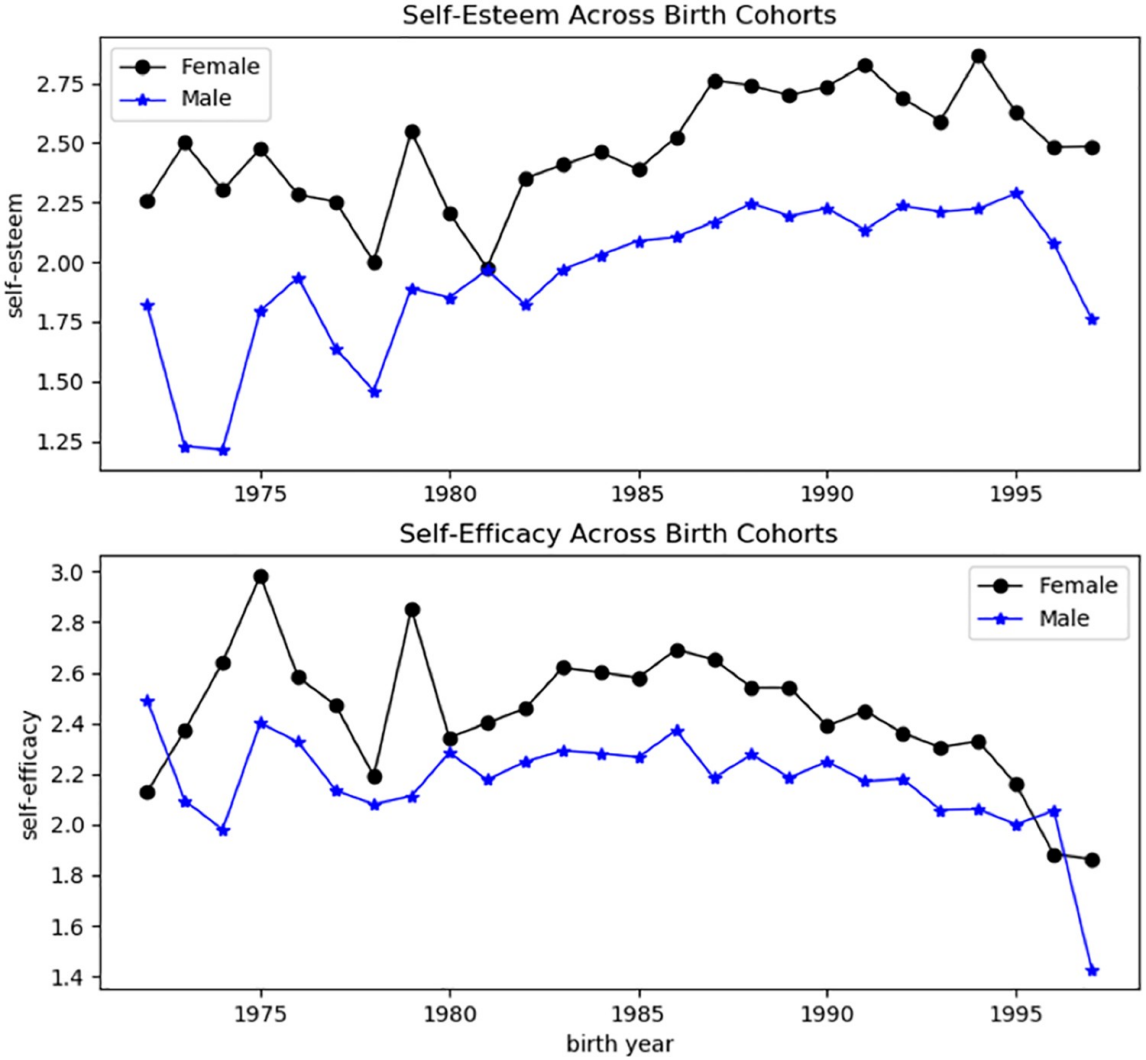
Gender differences in self-view across birth cohorts.

Finally, we create a dummy variable for each key word to indicate whether the word occurs in a resume. Furthermore, we add up the occurrences of the words in each of the two categories as variables *self-esteem* and *self-efficacy*. Besides, the length of self-view text is recorded as variable *description-length*, to measure the general richness of the content. As we can see from Table 4, women score higher in both self-esteem and self-efficacy but write a shorter self-view. We further check self-view across birth cohorts. To ensure the sample size of each cohort is larger than 100, we only select the subpopulation of birth year 1972 to 1997. Fig 1 shows that women of almost all cohorts score higher in both self-esteem and self-efficacy than men.

**Table 4:** Gender difference in global self-view.

Variables	Female	Male	Difference
self-esteem	2.583	2.043	0.541***
self-efficacy	2.449	2.201	0.248***
description-length (in 50-word)	2.65	2.97	-0.32***

Note:

***,**,* denote significance levels of 1%, 5% and 10%.

Although a large body of previous literature find that girls have distressingly lower self-esteem, some other meta-analysis find that the effect size for the gender difference in self-esteem is small or insignificant, and the effect size also depends on life stage. Besides, previous research finds a clear trajectory of systematic changes in self-esteem throughout the life-span age [24–25]. Moreover, it is not surprising that the disadvantaged groups have levels of self-view not lower than the advantaged groups. It is documented that Blacks and Chicanos have levels of self-esteem at least as high as those of Whites in many studies [20,26,27].

Our finding that women score higher in “global” self-view may also be related to the theories of “belief flipping” or disappearance of statistical discrimination against women who succeed getting into career track positions [28–29]. In line with the theories of “belief flipping”, our finding of differences in self-view favoring females makes sense if we note that our sample is from adults active in the labor market with on average 15.2 years of education and 8.99 years of work experience. These women are those who succeed getting into career track positions, and indeed those who climb to the top class, considering that the majority of population receive no more than junior high school education in a developing country like China. It is possible that women show higher self-view than men in this sample, due to self-selection. To shed light on this, we will do sensitivity analysis based on years of work-experience and along other dimension in part C of this section.

### B. Domain-specific self-view

In an effort to further reconcile the differences of our findings with the existing literature, we examine self-view across all 24 key words in Table 5. This is related to the “domain-specific” self-view literature. While most previous works examine “global” self-esteem, some other works explore into “domain-specific” self-esteem. For example, [30] find that although men score significantly higher than women on physical appearance, athletic, personal self, and self-satisfaction self-esteem, women score significantly higher than men in behavioral conduct and moral-ethical self-esteem, and no significant gender differences appear in academic, social acceptance, and family self-esteem. This finding can be extended to self-efficacy as well [31–32]. Our examination of self-view across 24 key words supports the conclusion from “domain-specific” self-view literature. As can be seen in Table 5, we find that men score significantly higher than women in teamwork, and problem-solving self-efficacy, women score significantly higher than men in moral-ethical, and social acceptance self-esteem, and no significant gender differences appear in independency self-esteem, and organizing ability self-efficacy. In summary, although women score higher than men in “global” self-view, men score higher than women in some self-view “domains”, which is consistent with literature.

**Table 5:** Gender differences in domain-specific self-view.

Variables	Female	Male	Difference
responsible	0.441	0.359	0.082***
active	0.256	0.201	0.055***
outgoing	0.288	0.182	0.106***
reliable	0.17	0.132	0.038***
hardy	0.136	0.128	0.007
optimistic	0.15	0.113	0.037***
enthusiastic	0.149	0.109	0.041***
conscientious	0.405	0.283	0.122***
independent	0.1	0.097	0.003
steady	0.081	0.083	-0.003
hard-working	0.111	0.083	0.028***
honest	0.077	0.073	0.004
excellent	0.067	0.065	0.002
team	0.406	0.426	-0.020***
learn	0.385	0.302	0.083***
communicate	0.41	0.338	0.072***
organize	0.188	0.187	0
coordinate	0.186	0.174	0.011**
adapt	0.249	0.188	0.061***
deal	0.102	0.092	0.009**
challenge	0.108	0.098	0.010**
problem-s	0.105	0.114	-0.009**
execute	0.138	0.134	0.003
stress	0.087	0.067	0.020***
N	10581	10012	

Note:

***,**,* denote significance levels of 1%, 5% and 10%.

There is little literature on self-view that uses other measures than self-esteem and self-efficacy. One exception is [23] that use five factors: Extraversion, Agreeableness, Conscientiousness, Neuroticism, and Openness to Experience. However, for the purpose of convincingness of self-view measures, we believe our 24-keyword method exhibits a more detailed picture of self-view than five-factor approach. Moreover, it turns out the 24-keyword method supports the results from global self-esteem and self-efficacy, and it helps reconcile the differences with the existing literature.

### C. Self-image or social signaling?

Even if it is possible that the disadvantaged group have levels of self-view at least as high as those of the advantaged group, and that our white-collar female sample fits theories of “belief-flipping”, one might still doubt if the result is a story of social signaling instead of a story of self-image. One might also argue that women perhaps spend more time preparing their resume. To this end, we calculate gender differences in self-view across different groups, based on whether they are fresh graduates, whether they are in female-dominated industries, and across groups of different marital status, and length of work experience.

Table 6 shows the results of gender differences for fresh graduates and workers with some work experience. It is clear that for fresh graduates, no significant gender differences exist in either self-esteem or self-efficacy. In comparison, significant difference in self-view favoring females exist in workers with some work experience. Table 7 further differentiates workers according to their previous job numbers. The results support those of Table 5. The more work experience, the larger the gender differences in self-view favoring females.

**Table 6:** Gender differences for fresh graduates and senior workers.

		Female	Male	difference
Self-esteem	Fresh graduate	2.72	2.43	0.29
	working	2.45	1.94	0.51***
Self-efficacy	Fresh graduate	2.46	2.45	0.01
	working	2.50	2.28	0.22***

Note:

***,**,* denote significance levels of 1%, 5% and 10%.

**Table 7:** Gender differences across work experiences.

		Female	Male	difference
Self-esteem	Fresh graduate	2.72	2.43	0.29
	Previous work number = 1	2.45	2.01	0.44***
	Previous work number = 2 or 3	2.65	2.17	0.48***
	Previous work number > 3	2.54	1.93	0.62***
Self-efficacy	Fresh graduate	2.46	2.45	0.01
	Previous work number = 1	2.11	1.90	0.21***
	Previous work number = 2 or 3	2.41	2.21	0.20***
	Previous work number > 3	2.65	2.28	0.37***

Note:

***,**,* denote significance levels of 1%, 5% and 10%.

Tables 6 and 7 together support the view that there is systematic change in self-view throughout career age [24–25], and the theories of “belief flipping” or disappearance of statistical discrimination against women who succeed getting into career track positions [28–29]. In fact, we observe that women evaluate selves more highly relative to men do when they gain more and more work experience.

One might also argue that women spend more time in preparing their resume. It is true that significant and pervasive levels of discrimination have been found against women in the labor markets [33–34]. However, there are studies that find a pro-female bias in callbacks in female-dominated or mixed occupations [35–36]. To reconcile conflicting findings, we compare self-view levels across industries with different levels of female domination. If women do spend more time in preparing their resume due to discrimination, we would see smaller effect size of favoring-female gender differences in self-view in more female-dominated occupations. Table 8 shows the results of gender differences for workers from different industries, with IT and real estate/ construction being traditional male-dominated industries, and finance and education being more female-dominated or mixed industries. The results show that no differences exist across industries in terms of gender differences. Therefore, we could say that it seems not true that women spend more time preparing their resume.

**Table 8:** Gender differences across industries.

	industries	Female	Male	difference
Self-esteem	IT	2.58	2.01	0.57***
	finance	2.54	1.97	0.57***
	real estate/ construction	2.61	2.06	0.55***
	education	2.63	2.06	0.57***
Self-efficacy	IT	2.72	2.34	0.37***
	finance	2.68	2.24	0.43***
	real estate/ construction	2.53	2.28	0.26***
	education	2.51	2.24	0.27***

Note:

***,**,* denote significance levels of 1%, 5% and 10%.

Finally, Table 9 shows the results of gender differences for married and unmarried subsamples. Note that our resume data from zhaopin.com can only be seen by potential employers, and is *not* available to the public. Therefore, no comprise is needed to be made for the marriage market signaling. The results that married subsample showing larger gender differences is consistent with the results from Tables 6 and 7. Married population are usually elder and have more work experience than unmarried population. Besides, it is understandable considering that in China, women do most of housework regardless of working or not. A married working female may have higher self-view than a male since she succeeds in managing both career and family.

**Table 9:** Gender differences across marital status.

		Female	Male	difference
Self-esteem	married	2.56	1.96	0.60***
	unmarried	2.67	2.32	0.34***
Self-efficacy	married	2.69	2.23	0.46***
	unmarried	2.64	2.32	0.33***

Note:

***,**,* denote significance levels of 1%, 5% and 10%.

In summary, our resume data from zhaopin.com can only be seen by potential employers, and is *not* available to the public, unlike LinkedIn. Therefore, social concerns, such as social-image or social signaling, is not the underlying mechanism in this study. On the contrary, we argue that the results reflect females’ true self-image. Besides, previous findings of Blacks and Chicanos having levels of self-esteem at least as high as those of Whites lend credibility to our finding that females can have higher self-view than men, at least in some domains. Moreover, we argue that the differences in self-view favoring females may be related to the theories of “belief flipping”, since women in our data sample are those who succeed getting into career track positions, and indeed those who climb to the top class given their much longer average years of education compared to that of national average in China.

## Conditional association of self-view with desired salary

In this section, we first perform baseline regression of desired salaries on gender indicators, using as controls human capital, marital status, enterprise type, living costs, and industry fixed effects to account for different job seeking procedures across industries. Then we explore the role of self-view in explaining desired salaries and gender differences. Finally, we use all keyword components of self-view to gain more insights on the structure of self-view itself and its connection to desired salaries.

### A. Baseline regression

Table 10 shows the results of OLS regression. The dummy variable of gender indicator, female, is significantly negative, meaning women propose lower desired salary than men. The first column is the baseline regression according to the standard Mincer equation [37] where education, work experience and its square term are included. After adding more control variables in column (2) and (3), we get larger adjusted R^2^, meaning these controls add explanatory power to the gender differences in desired salary. Specifically, column (2) shows that enterprise type, marital status, work status, and industry help explain the gender difference. Column (3) adds in hukou locality and the average housing price of a job seeker’s desired work city, to reflect local living costs. These two variables are not seen in previous literature, but well improve explanatory power in the sense that adjusted R-square increases substantially. Overall, column (1)–(3) shows that after controlling for variables like human capital, marital status, enterprise type, industries, living costs, women propose about 20% lower desired salary than men at 1% level of significance. We further perform Ordered Probit regression as a robustness check. We use the range category of desired salary as the dependent variable, since range category is the original data from the drop-down menu. The results, not reported here, support that women propose significantly lower desired salary than men, with different inclusions of controls.

**Table 10:** Regression results on desired salaries.

	(1)	(2)	(3)	(4)	(5)
female	-0.270***	-0.206***	-0.196***	-0.175***	-0.178***
	(0.008)	(0.007)	(0.007)	(0.007)	(0.013)
eduy	0.119***	0.093***	0.102***	0.091***	0.091***
	(0.003)	(0.003)	(0.003)	(0.003)	(0.003)
grad_985	0.234***	0.166***	0.125***	0.115***	0.115***
	(0.012)	(0.011)	(0.010)	(0.010)	(0.010)
grad_211	0.203***	0.148***	0.104***	0.091***	0.091***
	(0.012)	(0.011)	(0.010)	(0.010)	(0.010)
work-year	0.059***	0.056***	0.062***	0.060***	0.060***
	(0.002)	(0.002)	(0.002)	(0.002)	(0.002)
work-year^2^	-0.001***	-0.001***	-0.001***	-0.001***	-0.001***
	(0.000)	(0.000)	(0.000)	(0.000)	(0.000)
work-num	0.062***	0.065***	0.063***	0.058***	0.058***
	(0.002)	(0.003)	(0.003)	(0.003)	(0.003)
self-esteem				-0.065***	-0.068***
				(0.004)	(0.005)
self-esteem^2^				0.004***	0.003***
				(0.001)	(0.001)
self-efficacy				0.019***	0.020***
				(0.005)	(0.005)
self-efficacy^2^				-0.001	-0.001
				(0.001)	(0.001)
desc-length				0.044***	0.047***
				(0.005)	(0.006)
desc-length^2^				-0.001*	-0.001**
				(0.001)	(0.001)
female*esteem					0.007**
					(0.003)
female*efficacy					-0.001
					(0.004)
female *desc-length					-0.004
					(0.004)
Enterprise type, marital status, work status, industry fixed effects		Yes	Yes	Yes	Yes
hukou, housing price			Yes	Yes	Yes
N	20593	20593	17564	17564	17564
adj. R-sq	0.277	0.412	0.543	0.567	0.567

Note: standard errors in parentheses.

***, **, * denote significance levels of 1%, 5% and 10%.

### B. Mediator and moderator effects of self-view

Results reported in Table 11 use as dependent variables three dimensions of the job seekers’ self-view. Descriptive statistics of these variables were provided in Table 4. The results of Table 11 show that the coefficient of the dummy variable *female* is significantly different from zero in each regression. Thus, self-view can be a mediator to explain the gender differences in desired salary.

**Table 11:** Regression results on self-view.

	self-esteem	self-efficacy	desc-length (in 50-word)
female	0.449***	0.268***	-0.118***
	(0.032)	(0.030)	(0.031)
human capital, enterprise type, marital status, work status, hukou, housing prices	Yes	Yes	Yes
industry fixed effects	Yes	Yes	Yes
N	17564	17564	17564
mean of dependent variables	2.322	2.341	2.818

Note: standard errors in parentheses.

***, **, * denote significance levels of 1%, 5% and 10%.

Column (4) in Table 10 is to check the mediator effect of self-view on desired salary. As we can see, allowing for self-view variables in column (4) reduces the gender gap in column (3) (as captured by the coefficient of the *female* dummy) by about 0.02 log points, or 10% in relative terms. Besides, the coefficients of self-view variables are quite significant themselves, serving as good contributors. A one-point increase in the self-esteem decreases desired salary by 6.5%, with a decreasing marginal effect. A one-point increase in self-efficacy increases desired salary by 1.9%. For every 50-word increase in description length, desired salary increases by 4.4%.

To check the moderator effects, we add interaction terms of gender indicator and self-view. The results are reported in column (5) of Table 10. Self-efficacy and description-length do not moderate the relationship between gender and desired salary. Although there is a statistically significant coefficient for the interaction of gender indicator and self-esteem, the magnitude is too small to affect the slope of the relationship.

### C. Components of self-view

We further use each key word component as one dimension of self-view, to explore the inner relationships of the components and to check the robustness of previous results. S1 Table shows the correlation matrix of 24 key word components. Mostly they are weakly and positively correlated. The positive largest correlation coefficient is 0.46, and the largest negative correlation coefficient is -0.06.

Other than grouping into two categories of self-esteem and self-efficacy, a natural way to deal with the 24 dimensions is to apply principal component analysis (PCA). Table 12 presents the results. The first 7 components have eigenvalues larger than 1. Moreover, 17 components are needed to meet the requirement of explaining 80% of the variance. Therefore, roughly speaking, PCA is not very suitable. To formally measure how suited the data is for component analysis, we apply the Kaiser-Meyer-Olkin (KMO) test. The KMO statistic gauges the proportion of variance among variables that might be common variance. In other words, it measures sampling adequacy. The KMO statistic is 0.76, indicating a just middling sample adequacy. Therefore, we do not use PCA when analyzing the components of self-view. Instead, we directly use each component as a regressor.

**Table 12:** Principle components analysis.

	Eigenvalue	Cumulative variance
Comp1	3.07227	12.80%
Comp2	1.79488	20.28%
Comp3	1.21956	25.36%
Comp4	1.20222	30.37%
Comp5	1.08361	34.89%
Comp6	1.05919	39.30%
Comp7	1.03614	43.62%
Comp8	0.994185	47.76%
Comp9	0.950837	51.72%
Comp10	0.946628	55.66%
Comp11	0.928365	59.53%
Comp12	0.90796	63.32%
Comp13	0.901928	67.07%
Comp14	0.875069	70.72%
Comp15	0.848938	74.26%
Comp16	0.841828	77.77%
Comp17	0.796888	81.09%
Comp18	0.756796	84.24%
Comp19	0.74307	87.33%
Comp20	0.720348	90.34%
Comp21	0.695264	93.23%
Comp22	0.590378	95.69%
Comp23	0.534596	97.92%
Comp24	0.499056	100.00%

Table 13 reports the results of using all key word components of self-view as regressors. The first column has the same regressors as in column (3) of Table 5 except that the variables self-esteem, self-efficacy, description-length are replaced by the 24 key word components of self-view. As can be seen, 21 out of 24 variables have non-zero coefficients with significance levels at 1% or 5%, meaning almost every component has its own merit in explaining the desired salary. The second column includes interaction terms of these components and gender indicator. (The coefficients of the interactions are not reported to avoid the table being too lengthy.) The coefficients for the key words are generally unchanged, where 19 out of the 24 interaction terms are not different from zero at 10% significance level. This is consistent with the results for interaction terms when self-view was measured by self-esteem, self-efficacy and description length in Table 10, indicating no moderator effects. The third and fourth columns are for the female and male samples respectively. The coefficients are not much different from that of the first column, meaning the results from the first column are quite robust.

**Table 13:** Regression on all key word components of self-view.

		interaction	female	male
female	-0.181***	-0.197***		
	(0.007)	(0.012)		
resp	-0.007	0.005	-0.018*	0.006
	(0.007)	(0.011)	(0.009)	(0.012)
acti	-0.007	-0.015	-0.001	-0.014
	(0.008)	(0.013)	(0.010)	(0.014)
outgo	-0.038***	-0.043***	-0.038***	-0.038***
	(0.008)	(0.014)	(0.010)	(0.014)
relia	-0.069***	-0.077***	-0.062***	-0.075***
	(0.009)	(0.014)	(0.011)	(0.015)
hardy	-0.057***	-0.046***	-0.064***	-0.043***
	(0.010)	(0.015)	(0.013)	(0.016)
opti	-0.025**	-0.043***	-0.012	-0.040**
	(0.010)	(0.016)	(0.012)	(0.017)
enth	-0.021**	-0.038**	-0.010	-0.035**
	(0.010)	(0.017)	(0.012)	(0.017)
consc	-0.081***	-0.112***	-0.060***	-0.110***
	(0.008)	(0.012)	(0.010)	(0.013)
steady	-0.025**	-0.017	-0.029*	-0.017
	(0.012)	(0.018)	(0.015)	(0.019)
hard_w	-0.049***	-0.039**	-0.058***	-0.038**
	(0.011)	(0.017)	(0.013)	(0.018)
honest	-0.062***	-0.055***	-0.070***	-0.054***
	(0.012)	(0.018)	(0.016)	(0.019)
excel	0.043***	0.018	0.062***	0.022
	(0.013)	(0.019)	(0.017)	(0.021)
team	0.060***	0.080***	0.042***	0.077***
	(0.007)	(0.010)	(0.009)	(0.011)
learn	-0.024***	-0.038***	-0.016*	-0.036***
	(0.007)	(0.011)	(0.009)	(0.011)
commun	0.015**	0.014	0.016*	0.016
	(0.007)	(0.011)	(0.009)	(0.012)
organi	0.026***	0.031**	0.022*	0.031**
	(0.009)	(0.014)	(0.012)	(0.015)
coord	0.027***	0.037**	0.026**	0.032**
	(0.010)	(0.015)	(0.012)	(0.016)
adapt	-0.027***	-0.040***	-0.019*	-0.037***
	(0.008)	(0.013)	(0.010)	(0.014)
deal	0.012	-0.032**	0.048***	-0.033*
	(0.011)	(0.017)	(0.014)	(0.017)
challe	0.033***	0.037**	0.024*	0.038**
	(0.011)	(0.016)	(0.013)	(0.017)
probl	0.048***	0.066***	0.034**	0.067***
	(0.011)	(0.015)	(0.014)	(0.016)
execu	0.072***	0.082***	0.066***	0.081***
	(0.010)	(0.014)	(0.012)	(0.015)
stress	0.041***	0.045**	0.037**	0.047**
	(0.012)	(0.019)	(0.015)	(0.020)
N	17564	17564	9559	8005
adj. R-sq	0.563	0.564	0.554	0.530

Note: inclusion of controls is the same as in column (3) of Table 10. Standard errors in parentheses.

***,**,* denote significance levels of 1%, 5% and 10%.

## Conclusion

Desired salaries have the potential to crucially determine labor market outcomes, and gender differences in desired salaries can be an important cause of existing gender differences in labor market outcomes. When women propose a lower desired salary, they will be offered a lower salary: the process is self-fulfilling [38–39]. And a lower ongoing salary for females further undermines females’ salary expectation, which negatively impacts lifelong earnings [40]. One major challenge to better understand desired salaries is that they are difficult to observe in their natural environment. By using a large database of job seeking resumes in actual labor markets, we are able to explore the magnitude of and the determinants of gender differences in desired salaries.

We find gender differences in global self-view favoring females, and in some domain-specific self-view favoring males. In addition, we find that women do propose lower desired salary than men, after controlling for various factors such as human capital, marital status, industry fixed effects, etc. We further investigate the role of self-view and find it contributes to explain desired salaries, with modest mediator effect but little moderator effect on gender differences in desired salaries.

Our work on self-view, by using lexical approach, is somewhat limited in examining in full detail about potential mechanism underlying the phenomena. However, to reconcile some differences with the existing literature, we perform the gender differences of self-view across different groups, based on whether they are fresh graduates, whether they are in female-dominated industries, and across groups of different marital status, and length of work experience. We find that the gender differences reflect self-image, but not social signaling. In fact, our results show a very clear trajectory of increase in self-view of women relative to that of men throughout career span. Women score higher than men in self-view, and more so when they are married. And there’s not much difference in gender gap of self-view between the subsample from female-dominated industries and the subsample from non-female-dominated industries, meaning women polishing their resume is not the story here. Besides, previous findings of Blacks and Chicanos having levels of self-esteem at least as high as those of Whites lend credibility to our finding that females can have higher self-view than men, at least in some domains. Moreover, we argue that the differences in self-view favoring females may be related to the theories of “belief flipping”, since women in our data sample are those who succeed getting into career track positions, and indeed those who climb to the top class given their much longer average years of education compared to that of national average in China.

Our study, to our knowledge, is the first attempt to use data in actual labor markets to investigate gender differences in desired salaries, and also the first to use text data mining techniques in analyzing self-view in labor markets. The evidence on self-view in this study differs from previous lab evidence, adding to literature that emphasizes the importance of environmental setting in studying subjective views and behaviors in labor markets.

## Supporting information

S1 Table Correlation matrix of self-view. (DOCX)

## References

[1] Weichselbaumer D, Winter‐Ebmer R. A meta‐analysis of the international gender wage gap. Journal of Economic Surveys. 2005 7;19(3):479–511.

[2] U.S. Bureau of Labor Statistics. Household survey data from Employment and Earnings, 2017 annual averages. 2018. https://www.bls.gov/opub/ee/2018/cps/annavg37_2017.pdf

[3] Altonji JG, Blank RM. Race and gender in the labor market. Handbook of labor economics. 1999 1 1;3:3143–259.

[4] Bertrand M, Mullainathan S. Are Emily and Greg more employable than Lakisha and Jamal? A field experiment on labor market discrimination. American economic review. 2004 9;94(4):991–1013.

[5] Blau FD, Kahn LM. Changes in the labor supply behavior of married women: 1980–2000. Journal of Labor Economics. 2007 7;25(3):393–438.

[6] Flory JA, Leibbrandt A, List JA. Do competitive workplaces deter female workers? A large-scale natural field experiment on job entry decisions. The Review of Economic Studies. 2014 10 3;82(1):122–55.

[7] Raiffa H. The art and science of negotiation. Harvard University Press; 1982.

[8] Jackson LA, Gardner PD, Sullivan LA. Explaining gender differences in self-pay expectations: Social comparison standards and perceptions of fair pay. Journal of Applied Psychology. 1992 10;77(5):651.

[9] Hogue M, Dubois CL, Fox-Cardamone L. Gender differences in pay expectations: the roles of job intention and self-view. Psychology of Women Quarterly. 2010 6;34(2):215–27.

[10] Bursztyn L, Fujiwara T, Pallais A. 'Acting Wife': Marriage Market Incentives and Labor Market Investments. American Economic Review. 2017 11;107(11):3288–319.

[11] Leibbrandt A, List JA. Do women avoid salary negotiations? Evidence from a large-scale natural field experiment. Management Science. 2014 9 8;61(9):2016–24.

[12] Williams MJ, Elizabeth LP, Spencer-Rodgers J. The masculinity of money: Automatic stereotypes predict gender differences in estimated salaries. Psychology of Women Quarterly. 2010 3;34(1):7–20.

[13] Bowles HR, Babcock L, McGinn KL. Constraints and triggers: situational mechanics of gender in negotiation. Journal of personality and social psychology. 2005 12;89(6):951 10.1037/0022-3514.89.6.951 16393027

[14] Owens TJ. Self and identity. InHandbook of social psychology 2006 (pp. 205–232). Springer, Boston, MA.

[15] Heckert TM, Droste HE, Adams PJ, Griffin CM, Roberts LL, Mueller MA, Wallis HA. Gender differences in anticipated salary: Role of salary estimates for others, job characteristics, career paths, and job inputs. Sex roles. 2002 8 1;47(3–4):139–51.

[16] Brañas-Garza P, Capraro V, Ramírez ER. Gender differences in altruism on Mechanical Turk: Expectations and actual behaviour. Economics Letters. 2018 5 30.

[17] Rand DG, Brescoll VL, Everett JA, Capraro V, Barcelo H. Social heuristics and social roles: Intuition favors altruism for women but not for men. Journal of Experimental Psychology: General. 2016 4;145(4):389. 26913619 10.1037/xge0000154

[18] Capraro V, Sippel J. Gender differences in moral judgment and the evaluation of gender-specified moral agents. Cognitive processing. 2017 11 1;18(4):399–405. 10.1007/s10339-017-0822-9 28597324

[19] Capraro V. Gender differences in lying in sender-receiver games: A meta-analysis. Judgment and Decision Making. 2018 7 2;13(4):345–55.

[20] Rosenberg M. Conceiving the self. 1979 Basic, New York. 1979.

[21] Bandura A, Wessels S. Self-efficacy. W.H. Freeman & Company; 1997 2.

[22] Kanfer R, Wanberg CR, Kantrowitz TM. Job search and employment: A personality–motivational analysis and meta-analytic review. Journal of Applied psychology. 2001 10;86(5):837 11596801 10.1037/0021-9010.86.5.837

[23] McCrae RR, John OP. An introduction to the five-factor model and its applications. Journal of personality. 1992 6;60(2):175–215. 10.1111/j.1467-6494.1992.tb00970.x 1635039

[24] Kling KC, Hyde JS, Showers CJ, Buswell BN. Gender differences in self-esteem: a meta-analysis. Psychological bulletin. 1999 7;125(4):470 10414226 10.1037/0033-2909.125.4.470

[25] Twenge JM, Campbell WK. Age and birth cohort differences in self-esteem: A cross-temporal meta-analysis. Personality and Social Psychology Review. 2001 11;5(4):321–44.

[26] Wylie RC. The self-concept (Vol. 2). 1979.

[27] Jensen GF, White CS, Galliher JM. Ethnic status and adolescent self-evaluations: An extension of research on minority self-esteem. Social Problems. 1982 12 1;30(2):226–39.

[28] Fryer RG Jr. Belief flipping in a dynamic model of statistical discrimination. Journal of Public Economics. 2007 6 1;91(5–6):1151–66.

[29] Smith N, Smith V, Verner M. Why are so few females promoted into CEO and vice president positions? Danish empirical evidence, 1997–2007. ILR Review. 2013 4;66(2):380–408.

[30] Gentile B, Grabe S, Dolan-Pascoe B, Twenge JM, Wells BE, Maitino A. Gender differences in domain-specific self-esteem: A meta-analysis. Review of General Psychology. 2009 3;13(1):34.

[31] Pajares F, Graham L. Self-efficacy, motivation constructs, and mathematics performance of entering middle school students. Contemporary educational psychology. 1999 4 1;24(2):124–39. 10.1006/ceps.1998.0991 10072312

[32] Wilson F, Kickul J, Marlino D. Gender, Entrepreneurial Self-Efficacy, and Entrepreneurial Career Intentions: Implications for Entrepreneurship Education 1. Entrepreneurship theory and practice. 2007 5;31(3):387–406.

[33] Riach PA, Rich J. Field experiments of discrimination in the market place. The economic journal. 2002 11;112(483):F480–518.

[34] Sarsons H. Gender differences in recognition for group work. Harvard University 2015 12 3;3.

[35] Riach PA, Rich J. An experimental investigation of sexual discrimination in hiring in the English labor market. Advances in Economic Analysis & Policy. 2006 2 13;5(2).

[36] Booth A, Leigh A. Do employers discriminate by gender? A field experiment in female-dominated occupations. Economics Letters. 2010 5 1;107(2):236–8.

[37] Mincer J. Schooling, Experience, and Earnings. Human Behavior & Social Institutions No. 2.

[38] Major B, Konar E. An investigation of sex differences in pay expectations and their possible causes. Academy of Management Journal. 1984 12 1;27(4):777–92.

[39] Gasser M, Flint N, Tan R. Reward expectations: The influence of race, gender and type of job. Journal of Business and Psychology. 2000 12 1;15(2):321–9.

[40] O'shea PG, Bush DF. Negotiation for starting salary: Antecedents and outcomes among recent college graduates. Journal of Business and Psychology. 2002 3 1;16(3):365–82.

