# Supplementary material for: Gender differences in self-view and desired salaries: A study on online recruitment website users in China
Source: PLoS One. 2019 Jan 10;14(1):e0210072. doi: 10.1371/journal.pone.0210072 (PMC6328104; doi:10.1371/journal.pone.0210072)
Supplement: S1 Table — (DOCX) [file pone.0210072.s001.docx]

**S1 Appendix**

S1 Table Correlation Matrix of Self-View

|  | resp | acti | outgo | relia | hardy | opti | enth | consc | indep | steady | hard_w | honest | excel |
| --- | --- | --- | --- | --- | --- | --- | --- | --- | --- | --- | --- | --- | --- |
| resp | 1.00 |  |  |  |  |  |  |  |  |  |  |  |  |
| acti | 0.13 | 1.00 |  |  |  |  |  |  |  |  |  |  |  |
| outgo | 0.17 | 0.17 | 1.00 |  |  |  |  |  |  |  |  |  |  |
| relia | 0.08 | 0.09 | 0.07 | 1.00 |  |  |  |  |  |  |  |  |  |
| hardy | 0.20 | 0.19 | 0.20 | 0.08 | 1.00 |  |  |  |  |  |  |  |  |
| opti | 0.12 | 0.26 | 0.26 | 0.05 | 0.10 | 1.00 |  |  |  |  |  |  |  |
| enth | 0.12 | 0.20 | 0.30 | 0.04 | 0.23 | 0.05 | 1.00 |  |  |  |  |  |  |
| consc | 0.46 | 0.20 | 0.22 | 0.18 | 0.21 | 0.13 | 0.16 | 1.00 |  |  |  |  |  |
| indep | 0.03 | 0.02 | -0.01 | 0.01 | -0.01 | -0.01 | -0.01 | -0.02 | 1.00 |  |  |  |  |
| steady | 0.12 | 0.14 | 0.11 | 0.12 | 0.18 | 0.06 | 0.17 | 0.11 | -0.02 | 1.00 |  |  |  |
| hard_w | 0.05 | 0.10 | 0.07 | 0.05 | 0.06 | 0.08 | 0.09 | 0.10 | -0.03 | 0.08 | 1.00 |  |  |
| honest | 0.14 | 0.07 | 0.10 | 0.05 | 0.16 | 0.05 | 0.11 | 0.16 | 0.00 | 0.08 | 0.00 | 1.00 |  |
| excel | 0.01 | 0.03 | 0.00 | 0.01 | -0.01 | -0.02 | 0.01 | -0.03 | 0.03 | -0.01 | 0.03 | -0.01 | 1.00 |
| team | 0.18 | 0.19 | 0.14 | 0.07 | 0.11 | 0.12 | 0.14 | 0.14 | 0.10 | 0.10 | 0.05 | 0.06 | 0.07 |
| learn | 0.08 | 0.13 | 0.08 | 0.05 | 0.05 | 0.12 | 0.08 | 0.11 | 0.03 | 0.04 | 0.14 | 0.03 | 0.05 |
| commun | 0.15 | 0.08 | 0.13 | 0.00 | 0.06 | 0.07 | 0.08 | 0.08 | 0.08 | 0.03 | 0.04 | 0.06 | 0.08 |
| organi | 0.13 | 0.09 | 0.15 | 0.00 | 0.09 | 0.11 | 0.10 | 0.09 | 0.04 | 0.11 | 0.05 | 0.01 | 0.10 |
| coord | 0.11 | 0.05 | 0.08 | 0.01 | 0.02 | 0.11 | 0.05 | 0.06 | 0.06 | 0.05 | 0.04 | 0.03 | 0.09 |
| adapt | 0.14 | 0.17 | 0.21 | 0.09 | 0.17 | 0.13 | 0.18 | 0.16 | 0.02 | 0.12 | 0.03 | 0.04 | 0.01 |
| deal | 0.02 | 0.00 | -0.03 | 0.00 | -0.02 | -0.02 | -0.03 | -0.01 | 0.12 | 0.00 | -0.02 | -0.01 | 0.04 |
| challe | 0.07 | 0.09 | 0.07 | 0.04 | 0.11 | 0.11 | 0.08 | 0.07 | 0.02 | 0.03 | 0.06 | 0.05 | 0.02 |
| probl | 0.01 | 0.08 | 0.01 | 0.05 | -0.01 | 0.02 | 0.04 | 0.02 | 0.12 | -0.02 | -0.01 | -0.01 | 0.04 |
| execu | 0.03 | -0.02 | -0.04 | -0.01 | -0.06 | -0.02 | -0.04 | -0.05 | 0.06 | 0.01 | -0.04 | -0.01 | 0.06 |
| stress | 0.02 | 0.01 | 0.00 | -0.01 | 0.00 | 0.03 | -0.02 | -0.02 | 0.05 | -0.02 | -0.03 | -0.03 | 0.03 |

|  | team | learn | commun | organi | coord | adapt | deal | challe | probl | execu | stress |
| --- | --- | --- | --- | --- | --- | --- | --- | --- | --- | --- | --- |
| team | 1.00 |  |  |  |  |  |  |  |  |  |  |
| learn | 0.14 | 1.00 |  |  |  |  |  |  |  |  |  |
| commun | 0.25 | 0.13 | 1.00 |  |  |  |  |  |  |  |  |
| organi | 0.18 | 0.08 | 0.20 | 1.00 |  |  |  |  |  |  |  |
| coord | 0.20 | 0.08 | 0.36 | 0.40 | 1.00 |  |  |  |  |  |  |
| adapt | 0.18 | 0.17 | 0.10 | 0.16 | 0.10 | 1.00 |  |  |  |  |  |
| deal | 0.04 | 0.02 | 0.09 | 0.06 | 0.09 | 0.02 | 1.00 |  |  |  |  |
| challe | 0.12 | 0.08 | 0.07 | 0.03 | 0.05 | 0.13 | -0.01 | 1.00 |  |  |  |
| probl | 0.11 | 0.10 | 0.09 | 0.04 | 0.08 | 0.06 | 0.16 | 0.03 | 1.00 |  |  |
| execu | 0.08 | 0.04 | 0.12 | 0.08 | 0.13 | 0.01 | 0.04 | 0.00 | 0.05 | 1.00 |  |
| stress | 0.06 | 0.07 | 0.09 | 0.03 | 0.07 | 0.06 | 0.03 | 0.03 | 0.03 | 0.09 | 1.00 |
